# An adaptive annotation approach for biomedical entity and relation recognition

**DOI:** 10.1007/s40708-016-0036-4

**Published:** 2016-02-27

**Authors:** Seid Muhie Yimam, Chris Biemann, Ljiljana Majnaric, Šefket Šabanović, Andreas Holzinger

**Affiliations:** 1 TU Darmstadt CS Department, FG Language Technology, 64289 Darmstadt, Germany; 2 Josip Juraj Strossmayer University of Osijek Faculty of Medicine Osijek, Osijek, Croatia; 3 Research Unit HCI-KDD Institute for Medical Informatics, Statistics and Documentation Medical University Graz, Auenbruggerplatz 2, 8036 Graz, Austria

**Keywords:** Interactive annotation, Machine learning, Knowledge discovery, Data mining, Human in the loop, Biomedical entity recognition, Relation learning

## Abstract

In this article, we demonstrate the impact of interactive machine learning: we develop biomedical entity recognition dataset using a human-into-the-loop approach. In contrary to classical machine learning, human-in-the-loop approaches do not operate on predefined training or test sets, but assume that human input regarding system improvement is supplied iteratively. Here, during annotation, a machine learning model is built on previous annotations and used to propose labels for subsequent annotation. To demonstrate that such interactive and iterative annotation speeds up the development of quality dataset annotation, we conduct three experiments. In the first experiment, we carry out an iterative annotation experimental simulation and show that only a handful of medical abstracts need to be annotated to produce suggestions that increase annotation speed. In the second experiment, clinical doctors have conducted a case study in annotating medical terms documents relevant for their research. The third experiment explores the annotation of semantic relations with relation instance learning across documents. The experiments validate our method qualitatively and quantitatively, and give rise to a more personalized, responsive information extraction technology.

## Introduction and motivation

The biomedical domain is increasingly turning into a data-intensive science, and one challenge with regard to the ever-increasing body of medical literature is not only to extract meaningful information from this data, but to gain knowledge, insight, and to make sense of the data [1]. Text is a very important type of data within the biomedical domain and in other domains: it is estimated that over 80 % of electronically available information is encoded in unstructured text documents [2]. As an example in the medical domain, patient records contain large amounts of text which have been entered in a non-standardized format, consequently posing a lot of challenges to processing of such data and for the clinical doctor the written text in the medical findings is still the basis for any decision making [3, 4]. Further, scientific results are communicated in text form, consequently for the biomedical domain text is an indispensable data type for gaining knowledge [5].

Modern automated information extraction (IE) systems usually are based on machine-learning models, which require large amount of manually annotated data to specify the model according to the task at hand. Unfortunately, particularly in the medical domain, experts have obligations with higher priorities, thus it is very expensive and cumbersome to annotate a large number of training examples. In order to alleviate this problem, there is a need for an approach where human annotators are facilitated to annotate faster than the traditional way, in order to produce required annotations in less time.

A further complication, not only but especially in the medical domain, is the general difficulty with standardization in the light of genericity of the standard and specificity of the application scenario. While large-scale taxonomies ontologies exist both for the general [6, 7] and the medical domain (e.g. UMLS)^1^., their sheer size impose a high burden on anyone that tries to make knowledge in text explicit: annotators would have to learn these ontologies or at least relevant parts of these ontologies in order to properly carry out their task. Further, as [8] points out, ontologies are usually created in an author-centric fashion: without a particular application at hand, authors of ontologies have to discretize the space of things into concepts. This discretization, however might or might not be suitable and might or might not yield practical conceptualizations for a particular task or application. While automatically inducing ontologies from text [9] or other statistical methods to induce conceptualizations and taxonomies have the premise to alleviate the author-centricity by yielding a resource that neatly fits the domain as defined by the corpus, they are still hard to tune towards particular modelling goals of users, which might only find a small fraction of the textual material relevant for their task.

While we have seen tremendous efforts in the past years to standardize and link lexical taxonomies and ontologies^2^, there has not been a widespread use of such structured resources for the formal representation of the semantics of text. We attribute this to their excessive size and their author-centricity as outlined above, as well as to the lack of information for being able to assign their concepts to respective terms in unstructured text: just because e.g. all viruses are known in a database, it does not follow from this that it is possible to find their occurrences in text (e.g. because of ambiguous abbreviations, short forms and variants, idiosynchracies of the subfield etc.). Here, we propose a radical break with this traditional way of knowledge representation: instead, *users* should be able to choose their own set of categories per given task or problem, and thus should be able to grow their own local ontology without the need (but eventually with the possibility) of connecting it to existing upper ontologies, and users should *ground* their conceptualization in the respective *texts* of their current interest.

In this article, we tackle the extractions of entity mentions and their relations from biomedical texts, specifically from MEDLINE abstracts^3^, using a recent human-into-the-loop automation strategy that has not been applied in the medical domain before. Unlike named entity recognition (NER) systems on e.g. the news domain, entity recognition on medical domains comprises of extractions of technical terms in the broader medical and biological arena such as name of diseases, proteins, substances and so on, see e.g. [10, 11].

Such an automation approach is specifically very important for the medical domain, as a full manual annotation is extremely expensive. Medical professionals in turn, however, are willing to perform this task only diligently if it matches their current field of interest. The human-into-the-loop automation approach enables users to start the automation process without pre-existing annotations, and works by suggesting annotations as soon as the users have annotated a rather small number of documents. This annotate-little and predict-little strategy is deemed adequate for biomedical domains as it (1) produce quality annotation in a very short period of time, (2) the approach is adaptive in such a way that newly evolving concepts or entities will not be ignored by an old and static prediction classification model, and 3) the conceptualization (i.e. entity types and their typed relations) can be chosen and extended by the user during the annotation process. Thus, this human-in-the-loop approach follows the principles of the recently emerging cognitive computing paradigm that proposes more adaptive, iterative and interactive human-machine interaction [12, 13].

Note that while models trained on a small number of entity mentions cannot be expected to produce high-quality automatic labels, however their annotation suggestions might still be useful for the task at hand, in turn, help to produce more annotations in a short time that eventually improve the quality of the automatic labels.

We conduct three experiments to exemplify and evaluate our human-into-the-loop approach of entity mention annotation for the medical domain. In the first experiment (Sect. 5.1), we simulate the interactive machine learning approach by incrementally processing the BioNLP-NLPBA 2004 named entity annotated data set [14]. During the simulation, a classifier model is first trained on very few annotations and we measure the number and quality of correctly predicted annotations in the next chunk of the data, which subsequently is added to the training, simulating the annotation process. With this simulation, we can learn whether annotating very few documents already produces reasonable and faithful predictions so that it relieves users from annotating every document in the data set.

In the second experiment (Sect. 5.2), we put our approach to practice and apply it in a use case where medical professionals annotate documents in order to support research on their particular question of interest. Specifically, the task used for this study is focused towards the investigations of the causes of the B-chronic lymphocytic leukemia (B-CLL) on MEDLINE abstracts and users annotate terms with their respective entity classes with so-called span annotations, which means that annotators assign an entity label to a word or a subsequent set of words in the text. Here, we compare two setups where annotators are presented, or not presented with suggestions from the classifier in the interactive annotation interface. This experiment sets out to clarify whether medical professionals perceive our human-in-the-loop approach as appropriate and helpful in quantitative terms and in a qualitative assessment.

The third experiment (also in Sect. 5.2) extend this notion further: here, we focus on the relations between such entities, which is a more interesting type of knowledge from an application perspective, but also poses more challenging problem for incremental machine learning. Here, we let our medical expert annotate e.g. interactions between proteins or relations between antibodies and antigenes. We notice that the system quickly picks up on user-defined relations, and found that our medical expert had to define additional relations to relations given in a standard dataset in order to model her requirements.

The main contributions of this article are three-fold: first, we show how using the human-in-the-loop approach, we can outperform an approach that relies only on expert annotation without the human in the loop. Second, we demonstrate that even with a little amount of annotation, a good performance for annotation suggestion can be reached, resulting in a substantial annotation speedup. Third, we exemplify how the human-in-the-loop approach in text annotation allows the customization of entities and relation types for the user’s need. Part of this article was already presented in a shorter form in [15].

## Related work

This section gives a brief overview of related work in adaptive machine learning as well as named entity tagging and relation learning for the medical domain in general.

### Human into the loop

Automated machine learning algorithms work well in certain environments. However, biomedical data are full of probability, uncertainty, incompleteness, vagueness, noise, etc., which makes the application of automated approaches difficult, yet often impossible. Moreover, the complexity of current machine learning algorithms has discouraged medical professionals from the application of such solutions. There is also the issue of acceptability and provenance, cf. [16]: since their decisions might be life-critical, medical professionals will not accept automatic systems, even with high precision, which cannot justify the rationale for the automatic decision. While there exist rather simple learning algorithms (e.g. memory-based learning, [17]) that provide reasonable explanations (e.g. in form of similar examples or situations), they need more training data to reach the same performance level as more advanced and complex algorithms (e.g. deep learning [18]). However, most complex approaches fail to give interpretable reasons for their automatic classification, which calls for facilitation of training data creation, especially for sensitive and life-critical domains; a further drawback with complex machine learning approaches is that their training is done in epochs over the whole dataset and there is no straightforward way to add additional labeled examples to the model.

However, for increasing the quality of such approaches, the integration of the expert’s domain knowledge is indispensable. The interaction of the domain expert with the data would greatly enhance the whole knowledge discovery process chain. Interactive machine learning (IML) puts the human into the loop to enable what neither a human nor a computer could do on their own, cf. [1]. For this, only machine learning algorithms are suitable that support online learning. In this work, we will use a perceptron-based online learning algorithm to generate suggestions for manual text annotation in the medical domain. These annotations are subsequently used to generate better suggestions, as the model continuously updates based on human interaction with our annotation tool.

### Interactive and adaptive learning

Static machine learning assumes that the actual state of the “domain universe” can be sufficiently acquired by listing all available data sets at particular time. In the contrast, adaptive machine learning assumes the possibility that there might exist unrecorded facts at particular time, which can only be appear at some point in the future. This, however, is rather the standard situation than the exception: think e.g. of a recommendation system for an online shopping platform: if it was static, there would be no recommendations for any product that was launched after the system was set up. Authors of [19] address an industrial case study (tile manufacturing process) and found out that the classical machine learning setup faced difficulties such as (1) feedback is usually obtained after a process is completed, which might help the system, (2) some variables can change through time, and (3) error correction is always done after observation. The research by [20] on clustering a large number of documents using an interactive recommender system shows that users can sort documents into clusters significantly faster with an interactive recommender system than correcting the output of a static automated method. On top of simple user feedback in [21], such as accepting and rejecting suggestions, complex feedback like choosing the best features, suggestions for the re-weighting of features, proposing new features and combining features remarkably improve the system. Moreover, experiments in [22] examine the effect of allowing end users to do feature labeling, instead of annotating instances of training data: especially for small amounts of training, the feature labeling approach was shown to be effective. In our work, we do not incorporate feature labeling, but we will consider it in our future work.

### NER for medical domains

Recent years have seen a surge on biomedical text processing (see [23] for a survey), most of which rely on the GENIA corpus [24], which is a collection of biomedical abstracts. It is mainly annotated for linguistic structures such POS tagging and syntax annotation, semantic annotation of entities and so on [25, 26]. The work of [27] focuses on the automatic detections of multiple biomedical entities using a single-word classification approach in contrast to earlier works in the area focusing on single entity types such as proteins or genes. In this approach, they use features such as word attributes and contextual information. To alleviate the bottleneck of manual named entity annotation for medical texts, [28] have set up a crowdsourcing project on Amazon Mechanical Turk (www.mturk.com) to annotate three entity types. The research shows that using crowdsourcing is a viable alternative to annotate medical texts at scale for entity types that are understood by laymen like “medication”. However, for a more complex and fine-grained distinction that requires domain knowledge, medical professionals are required.

### Relation learning in the medical domain

EDGAR [29] is a natural language processing system that extracts information about drugs and genes relevant to cancer from the biomedical literature. The entities that EDGAR focuses on are genes, cells and drugs extracted from the MEDLINE abstracts. The system uses a statistical part-of-speech tagger for word class recognition and subsequently uses semantic and pragmatic information to construct possible relations. The entity relations (REL) task, a supporting task of the BioNLP shared task 2011 [30], deals with the extraction of two types of part-of relations between a gene or protein and an associated entity. The task focused on two specific types of object-component relations, that holding between a gene or protein and its part (domain, regions, promoters, amino acids, etc.) and that between a protein and a complex that it is a subunit of, namely protein-component and subunit-complex. The highest performing system achieves an F-score of 57.7 %. The work of [31] addresses the problem of automatic extractions of protein interactions from bioscience texts. Using graphical models and a neural network, it was possible to achieve a comparably high accuracy (64%) in extracting relations from biosmedical text. For training, a domain specific database of the HIV-1 human protein interaction database containing two types of interactions, protein interactions, and human gene knock-downs (replication interactions) was employed.

While described works constitute the state of the art of biomedical relation extraction, their level of performance is not sufficient for automatic processing. In our approach, we add the human in the loop to the equation, ensuring high accuracy on the specific relations of interest to our human annotator.

## Methodology

### Annotation learning

The development of large amounts of high quality training data at one shot is hard and even undesirable [32]. Instead, an interactive machine learning methodology is more applicable where the machine-learning model is enhanced not using the prevailing train-learn-evaluate technique, but improving the model in a more iterative fashion.

Interactive learning focuses on enhancing an existing machine-learning model based on newly acquired information, which is not possible in a classical machine learning setting. The benefit of interactive learning is many-fold, such as (1) the classifier model gets better and better as new training examples are added to the training data, (2) when there is a sudden change to the underlying data set, what is known as *concept drift*, the machine-learning model gets updated accordingly [33], and (3) it largely reduces the total annotation time required to annotate the whole dataset. Most importantly, such approach will (4) not require a pre-existing annotation dataset so that it is truly responsive and incremental, fully adaptive to the user’s need, and it makes such approach more affordable when integrated into a larger IE system. While it is possible to use pre-existing sets of labels for entities and their relations in interactive learning, this incremental methodology (5) also allows to define and extend these label sets at any point in time during the annotation. This might be an especially effective feature for avoiding the mismatch between the ontology or taxonomy of labels and the text collection.

As the machine-learning model can be enriched incrementally, applications employing this model will not be affected, as the system can still draw suggestions from the old model while building the new model. This approach overcomes the limitations where systems have to wait until full training and prediction cycles are completed, decreasing deployment time.

### The WebAnno annotation tool

To conduct our study, we have slightly extended WebAnno [34], which is a general purpose web-based annotation tool for a wide range of linguistic annotations. WebAnno allows to freely configure different span and relation annotations and is widely used for the creation of linguistic datasets in the natural language processing community as wenn as in the digital humanities.

WebAnno features an in-build automation mechanism as described in [35]. In this so-called “automation mode”, users can see automatic suggestions made by the system, where they can either accept or ignore. WebAnno [34] features a split-pane visualization, where annotation is performed in the upper pane by selecting text and choosing a label. In the lower pane, suggestions are displayed, which can be accepted and appear as annotations in the upper pane upon clicking on them, cf. Fig. 3.

We have extended WebAnno for this study to not only suggests span annotations but also relations between spans. While the span suggestion mechanism is very generic and relies on perceptron-based online learning of sequence tagging [36], the relation suggestion is restricted to the scenario where both spans are suggested and the relation between the terms of the two spans has been annotated before in the same or a previously annotated document. This restriction on relation learning produces highly precise suggestions, but fails to propose yet unseen relations.

### Medical NER tagging and relation extraction

Medical named entity mention recognition is a well-researched area with a large number of datasets used in competitions [14, 37–40]. These mainly focus on entity or mention and chunk detections and relation extraction. Unfortunately, biomedical annotation tasks are still challenging unlike other language processing tasks due to the fact that most of the annotations require highly experienced professional annotators, as discussed above.

To demonstrate the effect of interactive learning on biomedical entity tagging, we used thee BioNLP-NLPBA 2004 corpus and train a classifier using a rather generic sequence tagging system developed for German named entity recognition [41] based on CRF suite [42]. The system is highly configurable regarding features and data formats. For this study, we use basic standard features to characterize the text: Character and word features, which consists of the first and last character *n* grams (*n*=3) of the current token as affixes, considered in a time-shifted window of two tokens around the word token in focus. We also incorporated automatically induced part-of-speech (POS) tag clusters as features, which are based on the system by [43] trained on a medline 2004 dataset. For unseen tokens in the cluster, the pretree multi-purpose word classifier tool from the ASV toolbox [44] is used to approximate the unsupervised POS tags, which are induced following the principles of structure discovery [45]. Furthermore, word shape features that reflect capitalization and character classes (e.g. numbers vs. letters), were found to be relevant for biomedical mentions, as the shape of such entities often differs from non-entity tokens.

## Annotation problem use case

### Entity annotation

In this section, the use case of our medical research professionals is laid out. It focuses on understanding the interplay between risk factors and genetic presuppositions with a leukemia cancer.

B-chronic lymphocytic leukemia (B-CLL), a malignant hematopoetic neoplasm of B-lymphocytes (B cells), is the most common leukemia in the westernized world [46]. Yet, its risk factors and underlying mechanisms are still unknown. Some features of this malignancy, such as the incidence increasing with age and low proliferative capacity combined with impaired apoptosis (homeostatic cell death), categorize this disorder more as a chronic aging disease, than as a “real” leukemia, known to arise from the primary genetic defect and the subsequent block in immune cell differentiation [47]. On the other hand, accumulated evidence indicate that the pathogenesis of some commonly occurring cancers, such as breast, or colon cancer, as well as of some types of lymphomas (malignant neoplasms of the lymphoid tissue), can be explained by the complex interplay of age-related and lifestyle-related mechanisms, operating mainly through chronic inflammation and impaired insulin dependent metabolism, known as insulin resistance condition (decreased insulin action in target tissues followed by chronic hyperglycemia) [48–50].

Biological links towards cancerogenesis and lymphomagenesis go via impaired cell homeostasis mechanisms, including apoptosis and proliferation, as well as inter-cellular and intra-cellular signaling [51, 52]. Medical expert posed a hypothesis that the same risk factors and mechanisms stay also in the background of the pathogenesis of B-CLL. Exact evidence in the literature is absent. Literature search and reasoning could be demanding, because of the need to revealing many complex relationships between the numerous sets of entities and the syntagmatic constructs.

In order to alleviate the efforts of meaningful literature searching, we used the tool of adaptive annotation learning. Firstly, the medical expert prepared a set of selected abstracts, downloaded from the medline. Then, based on a limited number of specific medical entities, including cell, condition, disorder, gene, molecule, protein, molecular pathway and substance, she annotated the important structures throughout the entire text body and made them visible.

### Entity automation and relation copy annotator

In this second setup, we took datasets from the BioNLP 2011 shared task [40] (entity relations supporting task (REL)). Our tasks include (a) train a classifier for entity annotation, (b) correct suggestions provided by the classifier and when appropriate add new annotation to the dataset, and (c) create a relation annotation between the existing entity annotations. In addition to the relation types specified in the BioNLP shared task, our medical expert annotated additional relation types since the existing ones were not deemed sufficient for her research question. Table 1 shows the relation types specified at the shared task and our newly added relation types.

**Table 1:** Relation types from (a) the BioNLP shared task 2011 and (b) identified during the relation annotation process by our medical expert

	Descriptions
(a) Relation types from BioNLP 2011	
Equivalent	Two protein or cell components are equivalent
Protein-component	The protein-component is a less specific object-component relation that holds between a gene or protein and its component, such as a protein domain or the promoter of a gene.
Subunit-complex	Subunit-complex is a component-object relation that holds between a protein complex and its subunits, individual proteins
(b) New relation types	
Activator-reactor	Two proteins linked with the same reaction; the first one is responsible for starting the reaction and the second one responsible for its sustainability
Antibody–antigen	An immune protein with the ability to specifically bound the antigen, a foreign substance, and to neutralise its toxicity
Cell-marker	A set of surface proteins typical for a cell lineage or a stage of development
Cell-variant	The main cell lineage and the subtypes which are the parts of this larger cell family
DNA-transcript	DNA and its mRNA (messenger RNA) which translate the gene‘s message to a protein product
Ligand–receptor	Two proteins or molecules which can bind to each other because oft he complementarity of the binding site
Protein-variant	Two proteins with the similar structure and function

Already at this point, we can conclude that an adaptive approach to relation extraction is more adequate to the scenario of biomedical annotation and knowledge management: Only through an adaptive approach where users can freely addd new types of entities and relations it is possible to tune the explicified information towards the user’s needs: while the general-purpose setting in the BioNLP 2011 task has provided some useful relation types, it did not cover some of the relations of interest and a static approach would have left the user no choice but to disregard these or leave them in unstructured form.

**Fig. 1:**
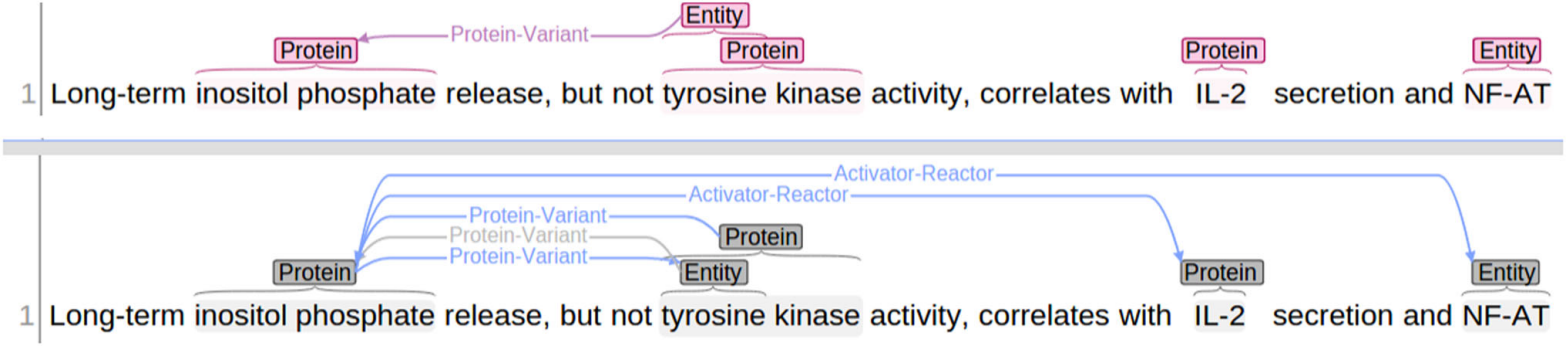
Relation copy annotator: upper pane: relation annotation by the annotator. *Lower pane*: relation suggestions that can be copied by the user to the *upper pane*

For rapid relation annotation, we have incorporated a relation copy annotator into WebAnno where relation suggestions are provided (at the lower pane in Fig. 1) as soon as annotators create relation annotations (in the upper pane in Fig. 1). This functionality has the following advantages: (a) more occurrences of the same relation are automatically suggested for the remaining parts of the document and for subsequent documents, and (b) an annotator can easily copy suggestions to the annotation view if the suggestions provided are correct. The impact of the relation copy annotation will be explained in the following section.

## Experiments and evaluation

### Simulating interactive learning

**Fig. 2:**
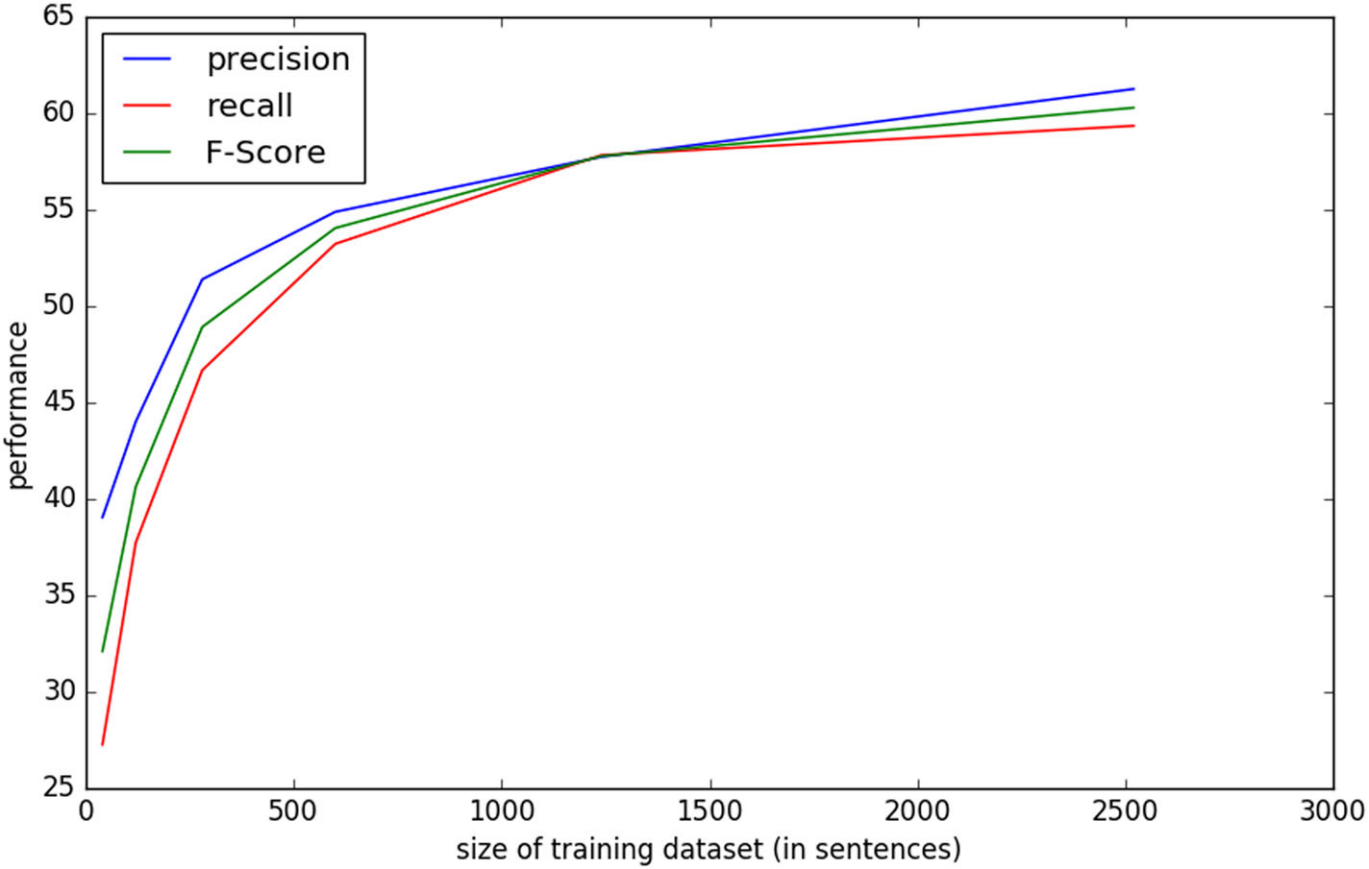
Learning curve showing the performance of interactive automation for BioNLP-NLPBA 2004 data set using different sizes of training data. (Color figure online)

In order to prove that interactive machine learning can yield a quality-annotated data set in a short training loop, we conduct our first experiment based on the BioNLP-NLPBA 2004 data set. The data set is divided into an increasing size of documents simulating interactive annotation. As it can be seen from Table 2 and Fig. 2, a (simulated) annotation of only 40 sentences already predicted an adequate amount of suggestions where users can quickly accept or modify and proceed to the next iteration. Aiming at maximizing F-score as the harmonic mean of precision and recall, we can clearly observe in Table 2 that, after simulated annotating of about 500 sentences, the gain in performance decreases, which implies that only annotating small portion of the sentences produces reasonable suggestions that are mostly acceptable by the annotator. Also, we can see that more annotations beyond 5000–10000 sentences are subject to diminishing returns, i.e. it takes an increasing number of annotations to achieve the same amount of relative improvements, the more annotations are used for training. In a human-in-the-loop setting, this can be detected during the process, and could be a sign for requiring more advanced features in the machine learning setup. This confirms our findings described in [53], where we have reached a speedup of factor 3 already with moderately accurate annotation suggestions.

**Table 2:** Evaluation result for the BioNLP-NLPBA 2004 task using an interactive online learning approach with different sizes of training dataset (in number of sentences) measured in precision, recall and F-measure on the fixed development dataset

Sentence	Recall	Precision	F-score
40	27.27	39.05	32.11
120	37.74	44.01	40.63
280	46.68	51.39	48.92
600	53.23	54.89	54.05
1240	57.83	57.74	57.78
2520	59.35	61.26	60.29
5080	62.32	64.03	63.16
10,200	66.43	67.50	66.96
18,555	69.48	69.16	69.32

### Automation and relation copy annotator

Using the BioNLP 2011 shared task dataset, we have conducted experiments constituting two phases, i.e. entity automation and correction as well as relation annotation and suggestion.

#### Entity automation

We have randomly selected 20 documents from the given training dataset (from a total of 780 documents) and train the in-built classifier of WebAnno (cf. Sect. 5.2). These documents contain 312 entity annotations and our classifier produced 687 annotation suggestions. Later we have presented the suggestions to our medical expert to re-annotate the documents using the suggestion. Our annotator produces a total of 752 entity annotations, which contains in addition to the protein and Entity annotations, a third type of entity called cell. Table 3 shows the performance of our automation system and expert annotator against the 20 documents (with gold annotations) form the BioNLP2011 REL shared task dataset.

#### Relation copy annotator

Once the entity annotation is completed, we have conducted relation annotation with the help of WebAnno copy annotator. The copy annotator produces relation suggestions in the same document where the source and target entity annotations as well as the covered texts match. The gold dataset contains 102 relation annotations while our annotator produces 397 relation annotations. Table 4 shows the average number of relation suggestions per document and across all documents.

**Table 3:** Machine learning automation and expert annotator performance for BioNLP 2011 REL shared task dataset

Mode	Annotator type	Recall	Precsion	F-score
Automation				
	Entity	61.94	49.31	54.91
	Protein	57.31	50.97	53.95
Expert				
	Entity	29.11	22.90	25.63
	Protein	71.94	59.28	65.00

We note that we are able to attain F-scores comparable to the state of the art, which validates out approach in comparison to previous approaches. More importantly, we expect a significant increase in performance when the system is used productively and can continuously extend its capabilities in long-running deployments.

**Table 4:** Analysis of relation suggestions. For a total of 20 randomly selected BioNLP2011 REL shared task documents, there has been a total of 397 relations annotated. In the process, the system produces on average 2.1 suggestions per relations and 19.85 suggestions per document. The last column shows an average number of relation suggestions across several documents

Docs	All	Rels	Perrel	Perdoc	Acrossdocs
20	397	193	2.1	19.85	0.18

### Qualitative Assessment

**Fig. 3:**
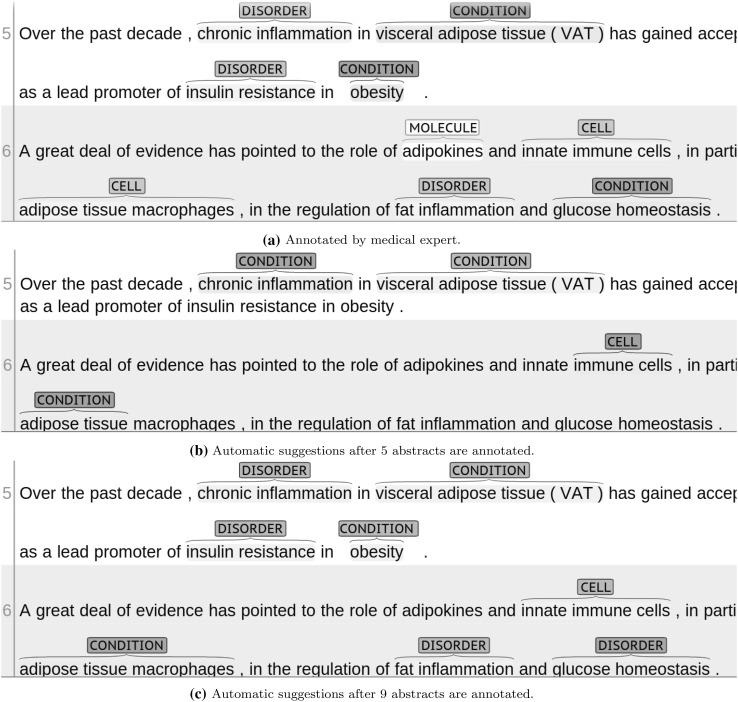
Automation suggestions using the WebAnno automation component after annotating 5 (**b**) initial response 9 (**c**) additional abstracts. Correct suggestions are marked in *grey*, while wrong suggestions are marked in *red*. **a** is the correct annotation by a medical expert. (Color figure online)

In addition to the quantitative experimental simulation done in Sect. 5.1, we have conducted practical annotation and automation experiments using a total of 10 MEDLINE abstracts that were chosen in the context of our use case described in Sect. 4, using WebAnno as described in Sect. 5.2. The experiment was conducted in two rounds. In the first round, medical experts have annotated 5 abstracts comprising a total of 86 sentences for specific medical entities as described in Sect. 4. Once the first round of annotations was completed, the automation was started using WebAnno’s automation component in order to provide initial suggestions. As displayed in Fig. 3, the automation component already suggests some entity annotations immediately after the first round. Using the automation suggestions, the expert continued annotating. After another 9 annotated abstracts that serve as training for the sequence tagging model, the quality and quantity of suggestions have again increased, see Fig. 3.

Qualitatively, annotators found that using the automation component, they perceived a significant increase in annotation speed. This confirms results in [53], where adaptive annotation automation in WebAnno can speed up the annotation process by a factor of 3 to 4 in comparison to a traditional annotation interface without suggestions. On a further note, the WebAnno tool was perceived as adequate and useable by our medical professionals, requiring only very limited usage instructions.

### Analysis of the automation and relation copy annotator

As it can be seen from Table 3, on one hand, the machine learning automation produces better performance on the general entity annotation types than our expert annotator. This indicates that the entities annotated in this dataset are very coarse level which should be re-annotated, specifically designed to meet domain and task requirements. On the other hand, our expert annotator outperforms the automation system on protein annotation types. This is because protein annotations are more specific and unambiguous to annotate.

The relation copy annotator behaves as expected, as shown in Table 4, where it is possible to produce more similar relation suggestion on the same document than across several documents. We can learn from this process that (1) the low number of relation suggestion across several documents (randomly selected from the dataset) indicates that we should employ human experts in the selection of documents which fit the domain of interest so that our system behaves as expected, and (2) a simple relation copy annotator fails to meet the need of producing adequate relation suggestions hence a proper machine learning algorithm for relation suggestion should be designed.

## Conclusion and future outlook

In this work, we investigated the impact of adaptive machine learning for the annotation of quality training data. Specifically, we tackled medical entity recognition and relation annotation on texts from MEDLINE, the largest collection of medical literature on the web. Identifying the need of entity tagging for applications such as IE, document summarization, fact exploring and relation extraction, and identifying the annotation acquisition bottleneck which is especially severe in the medical domain, we have carried out three experiments that show the utility of a human-in-the-loop approach for suggesting annotations in order to speed up the process and thus to widen this bottleneck. In the first experimental setup, we have used an existing BioNLP-NLPBA 2004 data set and run experimental simulation by incrementally processing the dataset to simulate the human in the loop. Using a generic sequence tagger, we showed that annotating very few sentences already produces enough correct predictions to be useful, suggesting that interactive annotation is a worthwhile enterprise from the beginning of an annotation project. In the second setup, we have engaged medical professionals in the annotation of medical entities in documents that were deemed relevant for the investigation of the cause of malignant B-CLL. The freely available WebAnno annotation tool (github.com/webanno) has been used for the annotation and automation process and annotators found that the adaptive annotation approach (1) makes it fast and easy to annotate medical entities, and (2) useful entity suggestions were already obtained after the annotation of only five medline abstracts, and suggestions subsequently improved tremendously after having annotated another nine abstracts, reducing the annotation effort. The third experiment extends the same notion to relation annotation, resulting in a graph of entities and their relations per document, which gives rise to a more formalized notion of medical knowledge representation and personal knowledge management.

On a larger perspective, our results demonstrate that a paradigm change in machine learning is feasible and viable. Whereas the mantra of the past has been ’there is no (annotated) data like more (annotated) data’ for supervised machine learning, suggesting large annotation efforts involving many human annotators, it becomes clear from our experiments that these efforts can be sped up tremendously by switching to an approach where the human can continuously improve the model by annotation while using the model to extract information, with the especially good news that the largest model improvements are achieved already very early in the process, as long as the domain is confined.

While such an adaptive approach to machine learning that factors in the user into the equation still calls for new evaluation methodologies to be assessed in all its aspects, it is deemed more adequate, more immediate and quicker deployable. It also fits better the shift towards an interactive, more natural, more adaptive, more contextualized and iterative approach under the umbrella of cognitive computing.

## Footnotes

1 https://www.nlm.nih.gov/research/umls/.

2 see http://www.w3.org/wiki/LinkedData.

3 www.ncbi.nlm.nih.gov/pubmed.
